# MiR-92b-3p is Induced by Advanced Glycation End Products and Involved in the Pathogenesis of Diabetic Nephropathy

**DOI:** 10.1155/2020/6050874

**Published:** 2020-03-06

**Authors:** Li-ping Wang, Jia-nan Geng, Bo Sun, Cheng-bo Sun, Yan Shi, Xiao-yan Yu

**Affiliations:** ^1^Department of Biobank, Clinical Medical College, Yangzhou University, Yangzhou, China; ^2^Department of Experimental Pharmacology and Toxicology, School of Pharmacy, Jilin University, Changchun, China

## Abstract

**Purpose:**

The current study aims to examine the effects of advanced glycation end products (AGEs) on the microRNA (miRNA) expression profile in the kidney tissues of rats.

**Methods:**

Wistar rats were randomly divided into three equal experiment groups: the AGE group, the RSA group, and the control group. The rats in the AGE group and the RSA group were administered with advanced glycation end products (AGEs) and rat serum albumin (RSA) via the tail vein, respectively, whereas the control group received PBS. Total RNA was prepared from the rat kidney tissues, and the miRNA expression profiles in different experiment groups were compared by microarray analysis. The expression levels of selected differential miRNAs were verified by RT-qPCR. Target gene prediction was conducted using algorithms such as TargetScan, miRanda, and PICTar. Functional analysis was performed to determine the putative biological roles of the validated miRNAs.

**Results:**

The microarray study revealed 451 upregulated and 320 downregulated miRNAs in the AGE group compared with the RSA group (*p* < 0.05). Seven miRNAs, including miR-21-5p, miR-92b-3p, miR-140-3p, miR-196a-5p, miR-181b-5p, miR-186-5p, and miR-192-5p, were screened and verified using RT-qPCR, of which, the change of miR-92b-3p was the most obvious according to the miRNA expression different multiple and *p* < 0.05). Seven miRNAs, including miR-21-5p, miR-92b-3p, miR-140-3p, miR-196a-5p, miR-181b-5p, miR-186-5p, and miR-192-5p, were screened and verified using RT-qPCR, of which, the change of miR-92b-3p was the most obvious according to the miRNA expression different multiple and

**Conclusion:**

The results of the current study suggested that miR-92b-3p could mediate AGE-induced development of renal abnormalities through targeting Smad7 in rats with DN.

## 1. Introduction

Diabetic nephropathy (DN) is a leading cause of death among diabetic patients and a major contributing factor to end-stage renal diseases [1]. Common pathological features of DN include the aggregation of extracellular matrix (ECM) proteins, the proliferation and hypertrophy of mesangial cells (MMCs), as well as the dysfunction of glomerular podocytes [2]. Patients with advanced DN can also develop glomerular lesions and proteinuria. Downregulated miRNA can inhibit MMCs proliferation in DN [3]. In recent years, the pathogenesis and treatment of DN have become a main focus of clinical research on diabetes and renal diseases. It is generally accepted that mutual reinforcement of metabolic dysregulation and hemodynamic abnormalities contributes to a vicious cycle of deteriorating renal pathologies in patients with chronic hyperglycemia [4, 5]. However, the exact mechanism for DN pathogenesis is extremely complex and remains poorly understood, which has hampered the development of effective diagnostic tools and therapies.

Advanced glycation end products (AGEs) are a structurally diverse group of glycated natural polymers generated by irreversible nonenzymatic reactions between the aldehyde groups of various reducing sugars, such as glucose and fructose, and the amino groups present in proteins and lipids. A wide range of studies have shown that the interaction of AGEs and the receptors for advanced glycation end products (RAGEs) can promote inflammatory response and the accumulation of oxidative stress, often through regulating the levels of various cytokines, hormones, and free radical species [6]. AGE levels are generally low in healthy individuals but gradually increase with aging. However, diabetic patients often exhibit abnormally high concentrations of AGEs due to sustained hyperglycemia, which increases the availability of circulating sugar substrates. AGE-induced crosslinking could result in the loss of function or even degradation of the modified protein substrates. Research has also suggested a link between DNA glycation and increased frequency of mutagenesis. The irreversible nature of AGE-promoted chemical modifications contributes to the so-called hyperglycemic memory effect, in which the glycated products cannot be eliminated by the restoration of normal blood sugar levels. Studies have demonstrated significantly higher renal AGE levels in diabetic animals and human patients [7–10]. It has also been shown that intravenous injection of AGEs can induce pathophysiological changes similar to those caused by DN in rats [11].

MicroRNAs (miRNAs) are a class of noncoding single-stranded RNA molecules with a typical length around 22 nucleotides. Since the discovery of the first miRNA in *Caenorhabditis elegans* in 1993 [12], the structural features and functional roles of these oligonucleotides have been elucidated in details. It is now well established that miRNAs play a key role in gene regulation by partially complementing with the 3′ untranslated region (3′-UTR) of their target mRNAs [13, 14]. It is estimated that approximately 30% of all protein-encoding genes in the human genome are modulated by miRNAs [15]. Recently, studies have suggested that miRNAs might also be mechanistically implicated in the early development of DN. For instance, Zhang et al. [16] reported that overexpression of miR-451 could lead to the downregulation of both MAP kinase kinase 3 (MKK3) and p38 mitogen-activated protein kinase (p38MAPK). Furthermore, an elevated miRNA-451 level was found to inhibit the proliferation of glomerular mesangial cells both in vitro and in vivo. In another study, miRNA profiling of a rat DN model revealed a panel of expressed miRNAs, among which downregulation of miR-21 was shown to have an inhibitory effect on mesangial cell proliferation and urine albumin excretion in the diabetic animals [17]. Wang et al. [18] found miR-377 to be highly expressed in laboratory-cultured human and mouse mesangial cells stimulated by the high concentration of glucose and transforming growth factor-*β*. Further investigation linked the overexpression of miR-377 to diminished cellular levels of superoxide dismutase and p21-activated kinase, as well as decreased fibronectin production, suggesting that miR-377 might be involved in DN pathogenesis by regulating ECM remodeling. Other miRNAs, including miR-21 [17], miR-192 [19], and miR-377 [18], have also been associated with DN-related pathologies.

In this study, miRNA microarray technology was used to observe miRNA expression profile change in the rat kidneys injected with AGEs, and the target miRNAs related to DN was screened. After validation of real-time PCR, the target genes of miRNAs were identified and verified by bioinformatics and biological methods, and it provides basis for the pathogenesis of DN and the search for therapeutic target. Based on the above findings, the current study aims to probe whether AGEs could affect the miRNA expression profile in rat kidneys. In addition, the study also seeks to examine the mechanistic roles of miRNAs in DN pathogenesis.

## 2. Materials and Methods

### 2.1. Preparation of Glycated Serum

The preparation of glycated serum was performed according to a previously described protocol with minor modifications [20]. AGE-rat serum albumin (AGE-RSA) was prepared by reacting RSA with 500 mmol/L of d-glucose, 100 U/mL of penicillin, and 100 U/mL of streptomycin under aerobic conditions for 10 weeks at 37°C. The control RSA reagent was generated under the same conditions without the addition of sugar. Both AGE-RSA and control RSA were extensively dialyzed in phosphate-buffered saline (PBS, pH 7.2) and then condensed in polyethylene glycol (molecular weight, 20,000) [11].

### 2.2. Animal Experiments

Ethical approval was obtained from the Experimental Animal Ethics Committee of Jilin University prior to the animal experiments (No. 20150305022; Date. 2015-03-05). A total of 15 healthy adult male Wistar rats, weighted between 220 and 250 g, were obtained from the Experimental Animal Center of Jilin University. The rats were fed a regular diet for one week and then randomly divided into three equal experiment groups, the control group (*n* = 5 rats), the RSA group (*n* = 5 rats), and the AGE group (*n* = 5 rats). The rats in the RSA group and the AGE group received daily intravenous injection of nonglycated and glycated sera, respectively, at a dose of 100 mg/kg. In contrast, the control group was administered with an equal dose of PBS. All rats were maintained under otherwise identical experimental conditions and weighed once a week throughout the entire experiment. Six weeks after the first injection, rat urine samples were collected twice with a 24 h interval in between the analysis of urinary albumin excretion (UAE) and the withdrawal of blood from main abdominal veins. The rats were then euthanized immediately, and their kidneys were harvested and stored at −80°C until use [21]. Total of six rats in the AGE and RSA groups were tested for microarray analysis by Biotechnology Co., Ltd (USA).

### 2.3. Cell Culture

The human embryonic kidney cell line (HEK293 cell) was maintained in DMEM (Gibco, Paisley, UK), 10% FBS (Gibco), 2 mmol/L glutamine, and penicillin-streptomycin 100 U/mL and kept in an incubator at 37°C in a humidified atmosphere containing 95% CO_2_. The cultures were passed every 2-3 days after brief trypsin treatment. Cells were plated onto glass coverslips 1 day prior to patch-clamp experiments.

### 2.4. Preparation of Total RNA and miRNA Microarray Analysis

The rat kidney tissues were stored in RNAlater Stabilization Solution (Thermo Fisher Scientific, PA, USA) at −80°C. Total RNA of the rat kidney tissues were extracted using the Trizol reagent and reverse transcribed to cDNA using the PrimeScript RT reagent kit according to the manufacturer's instructions. The quality of the extracted RNA was verified by measuring the A260/A280 ratio on a spectrophotometer and by agarose-formaldehyde gel electrophoresis. Approximately 5 *μ*g of the RNA was size-fractionated using a YM-100 Microcon centrifugal filter (Millipore, MA, USA) to isolate fragments shorter than 300 nucleotides, which were then 3′-extended with a poly(A) tail using poly(A) polymerase. An oligonucleotide tag was then ligated to the poly(A) tail to allow subsequent fluorescent dye staining. Overnight hybridization was conducted using a micro circulation pump (Atactic Technologies, TX, USA) and a *μ*Paraflo microfluidic chip (LC Sciences, TX, USA) [22]. Following RNA hybridization, detection was performed by circulating tag-specific Cy3 dye through the microfluidic chip. Images were collected on a GenePix 4000B microarray scanner (LC Sciences) and digitized by the Array-Pro Analyzer software (Media Cybernetics, MD, USA). The results were analyzed by first subtracting the background and then normalizing the signals using a LOWESS filter (LC Sciences) [23]. *p* < 0.05 was considered statistically significant.

### 2.5. Real-Time Quantitative Polymerase Chain Reaction (RT-qPCR)

MiRNAs and their putative target mRNAs were reverse-transcribed using specific primers on the ABI PRISM 7900 Sequence Detection System (Applied Biosystems, CA, USA). RT-qPCR was performed on the cDNA samples using the SYBR Premix Ex Taq II. The reaction conditions were as follows: 95°C for 30 s, followed by 40 cycles of denaturation at 95°C for 5 s and extension at 60°C for 34 s. The data were analyzed by the QuantStudio 7 Flex detection system. Cycle threshold (CT) values were analyzed by the comparative CT (ΔΔCT) method, and the relative amount of target mRNA (2−ΔΔ CT) was obtained by normalizing to glyceraldehyde-3-phosphate dehydrogenase (GAPDH) gene and U6 small-nuclear RNA. CT values were converted to fold changes. All experiments were performed in triplicate. *p* < 0.05 was considered statistically significant.

### 2.6. Bioinformatic Analysis

Putative miRNA-mRNA interactions were evaluated by TargetScan (version 7.2, http://www.targetscan.org/), miRanda (version 3.3a, http://www.microrna.org/microrna/home.do), and PITA (version 6, https://genie.weizmann.ac.il/pubs/mir07/mir07_data.html). The miRNA binding sites were predicted using the Ensembl BioMart web service (http://asia.ensembl.org/biomart/martview/). High-confidence miRNA-mRNA pairs were selected based on a combination of TargetScan context + score percentile >50, miRanda max energy < −20 kcal/mol, and PITA max energy < −10 kcal/mol.

### 2.7. Cell Transfection

HEK293 cells were transfected with miR-92b-3p inhibitor, inhibitor control, control-siRNA, Smad7-siRNA, or miR-92b-3p inhibitor + Smad7-siRNA using the Lipofectamine 2000 reagent (Invitrogen, USA) according to the manufacturer's instructions.

### 2.8. Dual-Luciferase Reporter Assay

To validate whether miR-92b-3p directly targets the 3′-untranslated region (3′-UTR) of Smad7, we performed a firefly luciferase reporter assay. We used TargetScan to predict the target genes of miR-92b-3p in the current study, and binding sites between Smad7 and miR-92b-3p were observed. The wild type (WT-Smad7) and mutated (Mut-Smad7) Smad7 were cloned into a pMIR-RB-Report™ dual-luciferase reporter gene plasmid vector (RiboBio, Guangzhou) according to the manufacturer's instructions. HEK293 cells were co-transfected with WT-Smad7 or Mut-Smad7 and miR-92b-3p mimic or mimic control using Lipofectamine® 2000 (Invitrogen, USA) as per the manufacturer's protocols. After transfection for 24 h, a dual-luciferase reporter assay (Promega, USA) was applied to determine the luciferase activity. Luciferase activity was normalized to the Renilla luciferase activity.

### 2.9. Western Blotting

Western blotting was performed according to a previously described procedure [24]. Kidney tissues and HEK293 cells were suspended in the lysis buffer provided with the radio immunoprecipitation assay kit (Beyotime Biotechnology, Shanghai, China) and homogenized by sonication. The resultant mixture was centrifuged at 3000 rpm for 10 min at 4°C. Proteins in the supernatant were separated on a 12% dodecyl sulfate-polyacrylamide gel, followed by transfer onto a polyvinylidene difluoride membrane. The membrane was then blocked with 5% skimmed milk, stained with 1 : 1000 Rabbit anti-Smad7 antibody (Abcam, UK) overnight at 4°C, and then with 1 : 5000 Goat anti-HRP antibody at 37°C for 1 h (ZSGB, Beijing, China). The membrane was developed using the BeyoECL Plus kit (Beyotime Biotechnology).

### 2.10. Immunohistochemistry

Immunohistochemistry was performed based on a previously described protocol [25]. Polyclonal rabbit anti-Smad7 antibody (Abcam, UK) was used as the primary antibody at a dilution level of 1 : 150. Polyclonal goat anti-HRP antibody (ZSGB, China) was used as the second antibody.

### 2.11. Statistical Analysis

The SPSS 20.0 statistical software was used for data processing. Counting data were shown as mean ± standard deviation. The statistical significance between two groups was assessed using the Student's 2-tailed *t*-test. One-way ANOVA followed by the Bonferroni–Dunn test was used for the comparison of more than two groups. *p* < 0.05 was considered significant.

## 3. Results

### 3.1. Examination of Urinary Secretion and Renal Pathological Changes

We began our study by first measuring the UAE of rats in the three experiment groups. UAE serves as a critical diagnostic indicator of the severity of DN and can be used for the staging of the disease. As shown in Table 1, there was no significant difference in the average volume of the collected urine between the three groups (*p* > 0.05). The average 24 h UAE of the AGE group was found to be higher than that of the control and the RSA groups (*p* < 0.05) (Table 1). The blood glucose level and the blood insulin level have no significant change in the AGE group compared with the RSA and control groups (Table 1). The experiment data thus suggested that the mice injected with glycated sera displayed some early signs of renal injury but could still be considered normoalbuminuric.

**Figure 1:**
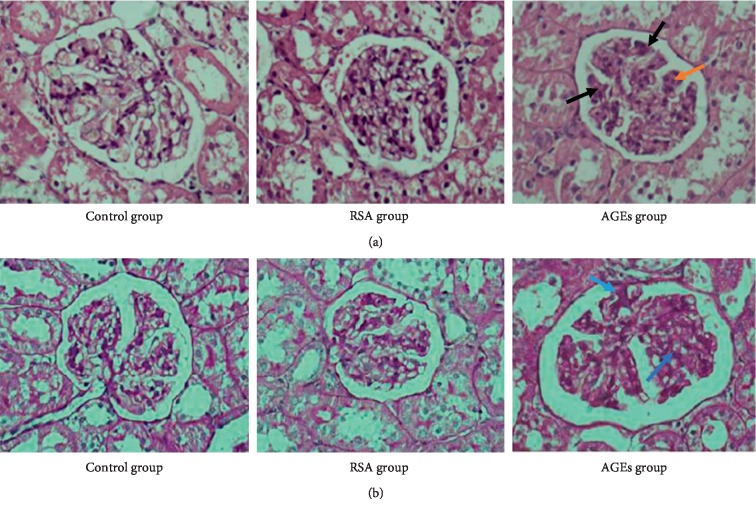
(a) HE staining of kidney tissues from the three experiment groups. (b) PAS staining of kidney tissues from the three experiment groups. The red arrow refers to the cell hyperplasia, the blue arrow refers to the ECM expansion, and the black arrow refers to the collapse of the glomerular capillary loops.

We next performed histopathological examination on the harvested rat renal tissues. H&E staining found the kidneys harvested from the AGE group to exhibit clear visual signs of mesangial cell hyperplasia and ECM expansion (Figure 1(a)). Furthermore, PAS staining also revealed collapse of the glomerular capillary loops in the AGE group. In comparison, neither the control group nor the RSA group displayed any of the abovementioned pathological changes (Figure 1(b)). The rats in these groups exhibited no capillary leakage, adhesion of glomerular tufts to Bowman's capsules, or mesangial expansion.

### 3.2. Identification and RT-qPCR Validation of Expressed miRNAs

**Figure 2:**
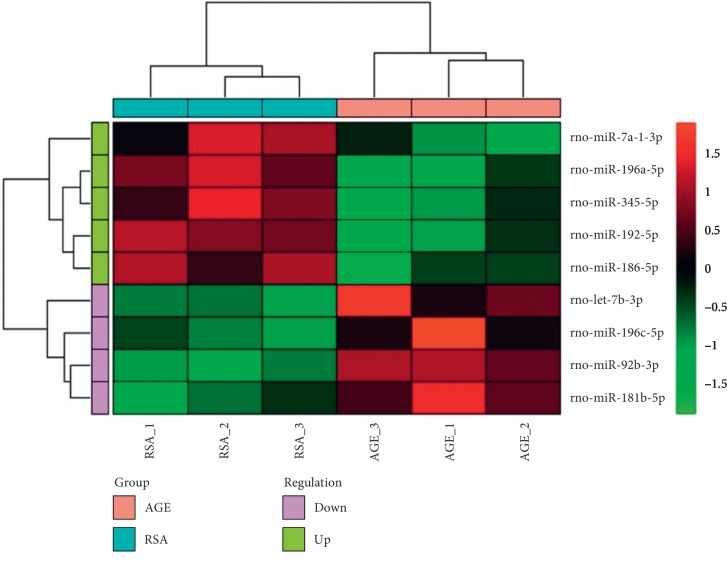
Heatmap denoting the expressed miRNAs between the RSA group and the AGE group.

**Figure 3:**
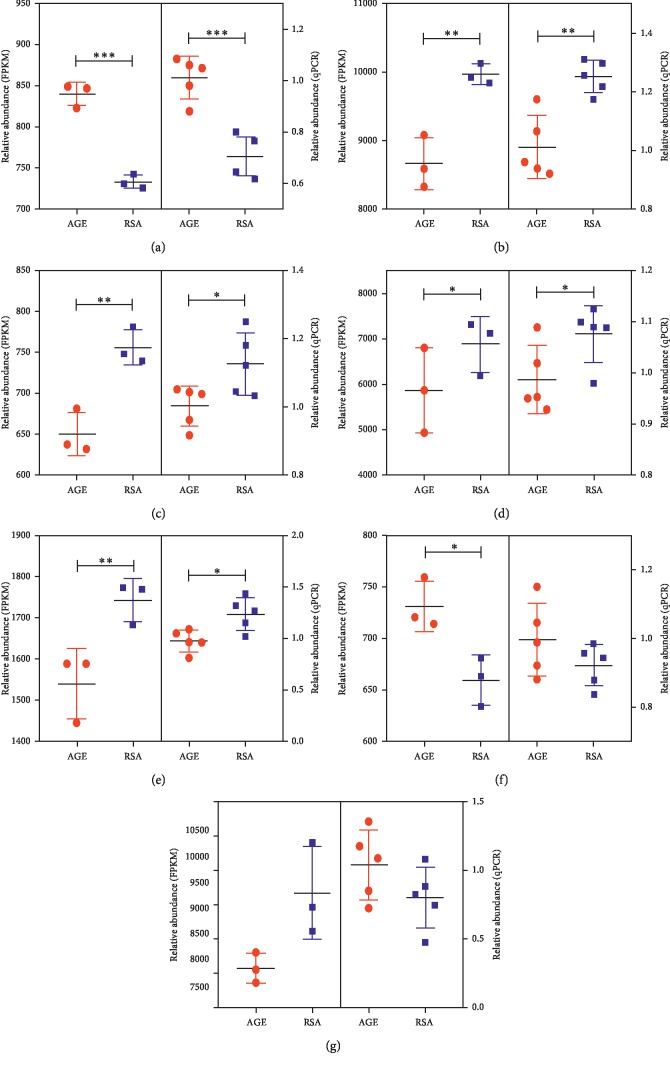
Validation of selected miRNAs by RT-qPCR. The left half of each plot shows the expression levels determined by microarray, whereas the right half indicates results from RT-qPCR analysis. (a) rno-miR-92b-3p. (b) rno-miR-192-5p. (c) rno-miR-196a-5p. (d) rno-miR-140-3p. (e) rno-miR-186-5p. (f) rno-miR-181b-5p. (g) rno-miR-21-5p.

Based on the results obtained from UAE rate measurement and histochemical examinations, we selected six rats each from the AGE group and the RSA group and analyzed the miRNA expression profiles in their kidney tissues. With the microarray data, we detected (1037) miRNAs and identified a panel of 771 candidates that showed expression between the two animal groups (*p* < 0.05). Among these miRNAs, a total of 451 miRNAs were upregulated and 320 miRNAs were downregulated in the AGE group compared with the RSA group. MiR-7d-3p, miR-196c-5p, miR-92b-3p, and miR-181b-5p were found to be upregulated, whereas miR-7a-1-3p, miR-196a-5p, miR-345-5p, miR-192-5p, and miR-186-5p were downregulated in the AGE group compared with the RSA group (Figure 2). Then, seven miRNAs, including miR-21-5p, miR-92b-3p, miR-140-3p, miR-196a-5p, miR-181b-5p, miR-186-5p, and miR-192-5p, were screened in this study according to miRNA expression differential multiple > 4 and *p* value < 0.005 and were verified by RT-qPCR. All miRNA expression trends detected by RT-qPCR were consistent with microarray results except miR-21-5p (Figure 3). Finally, we focused on miR-92b-3p for further functional analysis according to previously published research related to diabetic diseases.

### 3.3. Prediction of mRNA Targets of miR-92b-3p

The putative mRNA targets of miR-92b-3p were predicted by TargetScan, PICTar, and miRanda. A total of 265 target genes were identified by all three algorithms. The predicted mRNAs were shown by GO analysis to be involved in a diverse range of biological functions, including cellular metabolic processes, phosphorylation, cell proliferation and differentiation, cell apoptosis, as well as signal transduction. We speculated that miRNA-92-3p could be implicated in the pathogenesis of DN through targeting Smad7 (Table 2) based on a combination of TargetScan, miRanda, and PITA databases. Therefore, we subsequently focused on investigating the mechanistic role of Smad7 in DN pathogenesis.

### 3.4. Luciferase Assay Validates Smad7 as a Direct Target of miR-92b-3p

**Figure 4:**
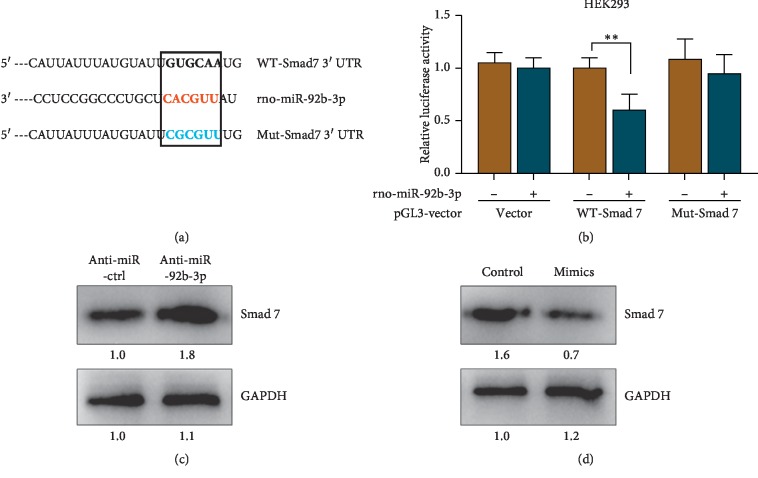
(a) The binding sites between Smad7 and miR-92b-3p. (b) Dual-luciferase reporter assay validated Smad7 as a direct target of miR-92b-3p. (c, d) The relative protein level of Smad7.

The data of TargetScan (http://www.targetscan.org) showed the binding sites between Smad7 and miR-92b-3p (Figure 4(a)). To reveal the potential binding sites of Smad7 and miR-92b-3p, luciferase reporter assay was performed. Compared with the cells co-transfected with MUT-Smad7 and miR-92b-3p mimic, the luciferase activity was significantly decreased in the cells co-transfected with WT-Smad7 and miR-92b-3p mimic (Figure 4(b)). The data indicated that miR-92b-3p directly targets Smad7. To further examine the effect of miR-92b-3p on Smad7, we transfected HEK293 cells with anti-miR-92b-3p or anti-miR-ctrl. Western blotting was performed to assess Smad7 protein levels. The results showed that Smad7 protein levels were increased in the HEK293 cells transfected with anti-miR-92b-3p compared with those transfected with anti-miR-ctrl (Figures 4(c) and 4(d)). Overexpression of miR-92b-3p by miR-92b-3p mimic transfection reduced the expression of Smad7 under NG conditions. Together, these results indicate that Smad7 is a direct target of miR-92b-3p.

### 3.5. Evaluation of Smad7 Expression in Rat Kidney Tissues

**Figure 5:**
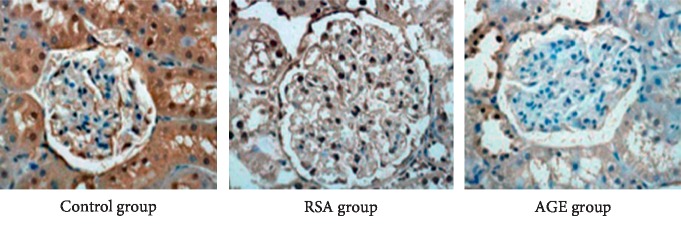
Immunohistochemical assessment of Smad7 expression in the kidney tissue from the three experiment groups. (a) Control group. (b) RSA group. (c) AGE group.

**Figure 6:**
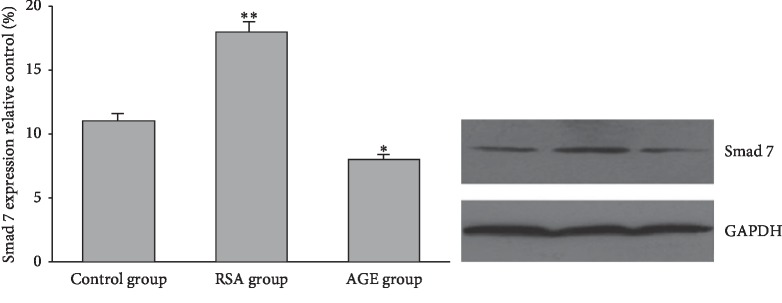
Western blotting analysis of Smad7 expression in the kidney tissue from the three experiment groups.

We next examined the renal expression of Smad7 in different experiment groups. Immunohistochemical staining revealed that the level of Smad7 in the kidney tissues of the AGE group was considerably lower than those in the control group and RSA group, as evidenced by the lighter staining of the cell nuclei (Figure 5). Consistently, western blotting also confirmed that Smad7 expression was significantly repressed in the AGE group compared with the other two groups (Figure 6). These results suggested that increased serum AGE levels were associated with both elevated expression of miR-92b-3p and downregulation of Smad7 in rat kidney tissues.

## 4. Discussion

In the current study, we investigated the regulatory effects of AGEs on miRNA expression in a rat DN model. Compared with the controls, the mice that received intravenous injections of glycated sera showed various DN-associated symptoms including albuminuria and glomerular hypertrophy. Microarray and bioinformatics analysis led to the identification of 451 upregulated and 320 downregulated miRNA candidates between the AGE and RSA groups. In particular, the expression of miR-92b-3p exhibited the most obvious change between the two groups among all miRNA candidates. Bioinformatics analysis suggested that miR-92b-3p could be involved in a variety of biological functions, including cellular metabolic process, phosphorylation, cell proliferation and differentiation, cell apoptosis, and signal transduction. In addition, several DN-related genes, such as TCF-21, TGIF1, Smad7, and TRAF3, were found to be the potential downstream targets of miR-92b-3p. Indeed, luciferase reporter assay confirmed that miR-92b-3p could interact with the 3′-UTR of Smad7. Furthermore, decreased renal expression of Smad7 was observed in the AGE group through both immunohistochemical and western blotting. Taken together, these results implied that miR-92b-3p could play an important role in mediating the renopathological effects of AGEs by targeting Smad7.

It is well accepted that AGEs contribute to DN pathogenesis by structurally modifying various biologically important macromolecules. Very recently, there is emerging evidence linking miRNAs to AGE-mediated pathological changes in diabetic kidneys. Li and colleagues reported that AGE treatment could stimulate the expression of miR-214 in THP-1 cells of the monocyte/macrophage lineage [26]. In addition, the authors demonstrated that the upregulation of miR-214 could mediate the inhibition of PTEN and cell apoptosis by activating RAGEs [26]. Similarly, Wu et al. showed that elevated AGE levels could repress the expression of miR-200b and miR-200c in human umbilical vein endothelial cells, which in turn would result in the dysregulation of several downstream target genes such as RhoA and ROCK. Consistent with these studies, our current finding that miR-92b-3p was significantly upregulated in AGE-injected rats offered further evidence that miRNAs are mechanistically involved in hyperglycemia-induced metabolic biochemical and metabolic alterations that lead to DN.

A series of recent studies have considerably furthered our understanding of the pathogenic role of Smad7 in DN [27–29]. The transforming growth factor-*β* (TGF-*β*)-Smad signaling pathway has been shown to be a key regulator of ECM homeostasis, a process closely associated with the development of the disease. Previous studies have found Smad7 to negatively modulate the TGF-*β* pathway by competing for receptor binding against Smad2 and Smad3 [30]. Furthermore, Smad7 has also been indicated to exert its inhibitory effect by inducing the ubiquitin-dependent proteasomal degradation of TGF-*β* receptor [31]. Based on these findings, it is not surprising that overexpression of Smad7 significantly mitigated renal fibrosis in a rat unilateral ureteral obstruction model [32]. Conversely, Smad7-deficient diabetic mice exhibited more severe renal fibrosis, albuminuria, and inflammation compared with those with a normal genotype [27]. The results of these studies were consistent with our observation of downregulated Smad7 expression in rat kidney tissues exposed to a hyperglycemic environment.

Smad7 has been shown to be a downstream target of several miRNAs previously associated with DN and other renal diseases. Mir-21 has been shown to aggravate diabetes-induced renal fibrosis by suppressing the expression of Smad7 and PTEN [33]. This was echoed by Chung et al.'s study, which found miR-21 mimics to be able to abolish the protective effect of Smad7 against renal fibrosis [34]. Interestingly, the authors also reported that the disruption of Smad7 could stimulate the expression of miR-21, suggesting a complex regulatory relationship between the two that could carry pathogenic implications for DN [34]. Smad7 has also been confirmed to be a downstream target of miRNA-192, and their interaction could promote epithelial-to-mesangial transition in tumor cells [35]. Similarly, inhibition of Smad7 by miR-92a could aggravate hypoxia/reoxygenation-induced myocardial injury and cell apoptosis [36].

Our current results identified miR-92b-3p as a new potential modulator of Smad7 and lent further support to the increasingly accepted notion that miRNAs could play important roles in mediating hyperglycemia-stimulated dysregulation of gene expression that contributes to pathological changes in renal tissues. At present, there are few new studies that used HEK293T cells to confirm Smad7 protein interacts with receptor-regulated Smads (R-Smads) to inhibit TGF-*β*/Smad signaling. In the future, kidney cell models will be used to further study whether the expression of miR-92b-3p and Smad7 is affected by AGE [30]. It is worth noting that we did not observe a significant difference between the average 24-h UAE of the AGE group and that of the other two groups. This implied that the alteration in miR-92b-3p expression occurred when the rats were still at the normoalbuminuric or microalbuminuric stage. Changes in miRNA expression profiles have been shown to be an early molecular event in the development of DN and have been suggested as a potential diagnostic biomarker. For example, in Jia et al.'s study, the level of miR-192 in renal tissues was found to be correlated with albuminuria and TGF-*β*1 expression even in normoalbuminuric DN patients [37]. The current study therefore suggested that miR-92b-3p could serve as a potential early indicator of DN development. Further investigation would be needed to determine the trend of miR-92b-3p in DN patients and to examine whether a predictive model could be constructed to identify early-stage DN in individuals that do not exhibit overt pathophysiological signs.

## Figures and Tables

**Table 1:** Average urinary volumes and 24-h UAE of the three experiment groups.

Group	*N*	Body weight (g)	Urinary volume (mL/24 h)	UAE (g/24 h)
Control group	5	307.19 ± 28.76	35.67 ± 16.97	9.02 ± 2.39^*∗*^
RSA group	5	314.11 ± 22.87	44.50 ± 18.38	8.73 ± 4.07^*∗*^
AGE group	5	288.12 ± 25.11	42.00 ± 19.80	13.38 ± 6.89

UAE, urinary albumin excretion; ^*∗*^AGE group *vs* control group, *p* < 0.05; AGE group *vs* RSA group, *p* < 0.05.

**Table 2:** Putative target genes of miR-92b-3p based on a combination of TargetScan, miRanda, and PITA predictions.

Transcript ID	Species ID	Gene ID	Symbol	miRBase ID	TargetScan	miRanda	PITA	Total
ENSRNOG00000000170	rno	64469	Slc30a4	rno-miR-92b-3p	1	1	1	3
ENSRNOG00000000459	rno	24967	Psmb9	rno-miR-92b-3p	1	1	1	3
ENSRNOG00000000520	rno	361814	Srsf3	rno-miR-92b-3p	1	1	1	3
ENSRNOG00000016700	rno	252856	Tcf21	rno-miR-92b-3p	1	1	1	3
ENSRNOG00000000694	rno	114027	Dao	rno-miR-92b-3p	1	1	1	3
ENSRNOG00000015906	rno	316742	Tgif1	rno-miR-92b-3p	1	1	1	3
ENSRNOG00000000894	rno	304244	Fry	rno-miR-92b-3p	1	1	1	3
ENSRNOG00000008145	rno	362788	Traf3	rno-miR-92b-3p	1	1	1	3
ENSRNOG00000001189	rno	59329	Sik1	rno-miR-92b-3p	1	1	1	3
*ENSRNOG00000018359*	*rno*	*81516*	*Smad7*	*rno-miR-92b-3p*	*1*	*1*	*1*	*3*
ENSRNOG00000001285	rno	29693	Atp2a2	rno-miR-92b-3p	1	1	1	3
ENSRNOG00000001314	rno	304334	Fam20c	rno-miR-92b-3p	1	1	1	3
ENSRNOG00000001460	rno	308178	Vps37c	rno-miR-92b-3p	1	1	1	3
ENSRNOG00000001688	rno	304071	Sim2	rno-miR-92b-3p	1	1	1	3
ENSRNOG00000018404	rno	292023	Aars	rno-miR-92b-3p	1	1	1	3

## Data Availability

All data used in this article can be accessed from the corresponding author.
